# Cholera in Rutherford County, Tenn.

**Published:** 1849-09

**Authors:** 


### THE WESTERN JOURNAL.

THIRD SERIES.—VOL. IV.—NO. III.

LOUISVILLE, SEPTEMBER 1, 1849.

### CHOLERA IN RUTHERFORD COUNTY, TENNESSEE.

This disease has not yet been epidemic in this county, but some cases have occurred under circumstances which appear to us instructive.

During the time when the epidemic was most fatal in Nashville, a man of dissipated habits, who had been to that city, was attacked with the disease on his return home, and died. A number of the persons who attended him in his illness, and assisted at his burial, were seized with the complaint in a few days, and also died. The disease did not afterwards spread.

A man, residing in another quarter of the county, contracted the seeds of the disease on a steamboat, and died in a few days after reaching his home. No other case occurred in his neighborhood.

These two cases are an offset to each other. The first favors the idea of contagion; but the other shows that something else is necessary to the propagation of the disorder than the introduction of a case into a neighborhood.

On the 17th of July, the wife of a laboring man, living thirty miles from Nashville, on the Milton turnpike, was attacked, and died after fifteen hours illness, with well-marked symptoms of cholera. Her son, aged 17 years, was attacked shortly afterwards, but his symptoms being attended to at an early period, he recovered after a hard struggle.

In a week from this date, a negro woman, at Epps Matthews, on the same road, died of cholera; and in a week after her death, her husband was attacked and died. Two days subsequently, William Matthews, the master of the last named slave, residing within a few hundred yards of his brother, was seized with violent symptoms of the disease, and died after an illness of thirteen hours. On the same night a second negro died at the farm of Epps Matthews. The families, after this, deserted their houses, and no new cases occurred among them; nor did the disease spread through the neighborhood.

On the farm of J. B. Rucker, two miles south of the Matthews', a negro woman was attacked with the disease, and died on the 8th of August, five days after the death of W. M. Mr. Rucker at once removed his negroes from their houses and built tents for them in the woods, where they have remained, now two weeks, entirely exempt from the malady.

An intemperate man, residing two miles east of Mr. R.'s, was affected with alarming symptoms on the 13th of August, but by timely medical aid, he was relieved. On the 18th, a negro woman, at Dr. Rucker's, close by, was attacked, and, a few days before, three fatal cases occurred among negroes in the neighborhood of Murfreesborough, four miles distant. None of these cases could be traced to intercourse among the affected.

In nearly all the cases, it appeared, on questioning the subjects, that diarrhea had preceded the violent symptoms from two to three days. In every instance, thus far, the spread of the pestilence has been arrested by the removal of the people from their houses.

The character of these houses merits a passing remark. They are, without an exception, small, low, ill-ventilated and filthy, with floors on the ground, and consequently damp in wet weather; in a word, precisely the abodes in which cholera would be expected to make its appearance. Whether from these wretched hovels it will ascend to the comfortable dwellings of the masters of the slaves, remains to be seen. Up to the present day (21st August) no such tendency is observable.

The weather has been such as has generally attended epidemic cholera in this country. The east wind has prevailed almost uninterruptedly, and with it there have been frequent and most abundant rains. During the month of July, few days passed without a shower, and on one or two the full average monthly mean is computed to have fallen. A state of weather more exactly resembling that which preceded the irruption of cholera at Lexington, in 1833, could not be imagined. The thermometer frequently rose as high as eighty-five degrees in the shade; but for the most part the temperature was rather below than above the summer mean.

The first case was excited by indigestible food, and the subject was a favorable one for cholera—a gross feeder and intemperate. Mr. M. is the only other white person who has died, and respecting him it is interesting to remark, that nearly all his father's family have died young, several of them of bowel complaints, and that his residence was near a large pond, into which all the water runs that falls upon his barn and stable yards. Besides this, south of his house, and within a stone's throw of the house of his brother, where two fatal cases occurred, there is a second pond, on the margin of which the stables and a cotton-gin are built. The stench from these ponds was such as to attract the attention of all passers-by, before cholera broke out in these families.

If we analyze the testimony afforded by these cases of cholera we shall find in it something that favors the contagiousness of the disease, but much more that goes to prove it to be of local origin. Most of the subjects resided on public highways, and it is impossible to say that the poison of the pestilence was not brought to them by some traveler from Nashville, where the epidemic was prevalent; but they also, as we have seen, lived in damp, confined, dirty houses, in the neighborhood of ponds, and amid all the conditions favorable to the development of malaria. It cannot be shown that there was any intercourse between the inmates of the families in which the disease successively appeared; and it is certain that it was not communicated to those who nursed the sick, or aided in the burial of the dead. That it ceased so suddenly on the desertion of their homes by the affected families, is an instructive fact. The only other remark we shall make is, that none of the patients who died were seen by a physician until they were in collapse, and that uniformly when the premonitory symptoms have been regarded the disease has proved easily manageable.

